# Color Compensatory Mechanism of Chromatic Adaptation at the Cortical Level

**DOI:** 10.1177/20416695221105538

**Published:** 2022-06-08

**Authors:** Hitomi Shimakura, Katsuaki Sakata

**Affiliations:** 43754 Shiseido Co., Ltd MIRAI Technology Institute, Yokohama, Japan; Department of Fine Arts, 12841 Joshibi University of Art and Design, Sagamihara, Japan

**Keywords:** neutral point, chromatic adaptation, temporal characteristics, compensation mechanism

## Abstract

Reportedly, some chromatic adaptations have extremely short temporal properties, while others have rather long ones. We aimed to dynamically measure the transition of a neutral point as an aftereffect during chromatic adaptation to understand the temporal characteristics of chromatic adaptation. The peripheral retina was exposed to a yellow light to progress color adaptation, while the transition of a neutral point was measured at the fovea. In Experiment 1, the aftereffect had initially progressed but subsequently recovered despite ongoing chromatic adaptation and regardless of the retinal exposure size, suggesting that the adaptation mechanism at the cortical level continues to readjust the color appearance based on daylight conditions. Experiment 2 was similar to Experiment 1, except that it included participants of varying ages. Older eyes behaved in a homologous manner with younger eyes in Experiment 2, albeit quantitative differences. Regardless of age, similar recalibration of neutral points shifted by color adaptation suggests the color compensation function in older eyes may not change due to long-term chromatic adaptation by optical yellowing. In conclusion, the chromatic adaptation mechanism at the cortical level readjusts color perception, even in younger eyes, according to the daylight neutral point. This daylight information may be stored in the neural mechanism of color vision.

## Introduction

Color vision has a trichromatic characteristic due to the presence of three types of cone cells for color perception in the retina. However, the characteristics of color perception are based on four cardinal axes at the post-photoreceptor level, such as the retinal ganglion cells or lateral geniculate nucleus. In addition, at the cortical level, neurons display selectivity for four cardinal directions (Danilova & Mollon, 2012; Krauskopf et al., 1982), and in recent years, intermediate directions have also been clarified (Elliott et al., 2012; Kuriki et al., 2015).

As the outputs of the three-cone mechanism can be calculated from the presented wavelength using the sensitivity curves of the three types of cones, it is important to acknowledge higher color-processing stages to understand how the sensitivity of the three cones is adjusted (Kraft & Werner, 1994; Wuerger et al., 2010).

The persistence of the aftereffects of chromatic adaptation indicates that sensitivity to identical light has been recalibrated, and this recalibration has been reported for both relatively brief and longer adaptations. The unique yellow color after chromatic adaptation by colored filters showed the rapid changes at first and then the slow changes, which suggested the existence of two adaptation processes, of which time courses showed an initial rapid change and a later slow one (Engel et al., 2016). The habitual wearing of colored filters caused changes in the appearance of color, which persisted for 1–2 weeks (Neitz et al., 2002). Furthermore, there is a central adaptation process at the cortical level with a long temporal characteristic, which is adapted by color light input from both eyes (Shimakura & Sakata, 2019). These studies show the possibility of the chromatic adaptation mechanism not specific to retinal location, namely the mechanism of long-term chromatic adaptation existing at the cortical level. As a result of this recalibration, an aftereffect occurs, which is defined as a shift in the achromatic point or neutral point like the unique yellow color which is neither reddish nor greenish, and the achromatic point shift depends on the magnitude of the color contrast adaptation (Delahunt et al., 2004; Webster & Wilson, 2000).

Moreover, there was no difference in the strength between 10 and 60 min of exposure to chromatic adaptation stimulus, suggesting that real-time recalibration for the stimulus history was completed (Tregillus & Webster, 2014). Namely, the magnitude of the aftereffect did not show accumulation regardless of the adaptation duration; that is, adaptation did not show much carry-over effect despite continuous sensory input of adaptation light. This suggests that the aftereffect continues to disappear despite continuous adaptation. To shed light on this continuous disappearance of the aftereffect, it is necessary to measure it during the chromatic adaptation. This requires that the adaptation area on the retina is separated from the area used for the measurement of its aftereffect.

Here, we measured the temporal characteristics of the aftereffect of long-term chromatic adaptation by partially exposing the retina to the adaptation light to better understand the chromatic adaptation recalibration process. The fovea cannot be exposed to high intensity adaptation light for a long period, taking into consideration fatigue in the participants. Therefore, we irradiated the periphery of the retina with an adaptation light of an intensity that could measure the aftereffect, measured in the fovea as a shift of the achromatic color point. Thus, the purpose of this study was to comprehensively elucidate the temporal characteristics of the aftereffects of chromatic adaptation during the progress of color light adaptation by exposing the peripheral retina to adaptive light and measuring the aftereffects of chromatic adaptation in the fovea.

There is a certain possibility of direct neural interaction between the adaptation light exposure area and the aftereffect measurement area in the fovea. However, in addition to neurophysiological evidence that the central and peripheral retinas have extremely different cone densities (Elsner et al., 2017) and that their mechanisms of lateral inhibition by horizontal cells are also different (Tuten et al., 2017), psychophysical findings have revealed that the peripheral retina is more sensitive to the L cone input than to the M cone input and that it is more sensitive to the S cone input than to the L + M cone input. It has also been reported that the area of spatial summation of the brightness detection threshold (Ricco’s area) increases as the degree of retinal eccentricity increases. Therefore, since the peripheral retina and fovea reportedly differ greatly, complex neural mechanisms are likely required for their interaction. Thus, the effect of retinal peripheral color adaptation on color appearance in the fovea could be considered as an interaction occurring at higher levels of processing. Moreover, considering the size of the receptive field of retinal ganglion cells around the fovea (Raninen & Rovamo, 1987), we deemed that no direct interaction on the retina had occurred, at least in the 6° region around the fovea.

To this end, in Experiment 1, the aftereffect of chromatic adaptation in the fovea was measured during adaptation in the peripheral retina to understand the temporal characteristics of chromatic adaptation. To explore the effect of adaptation magnitude, we employed two sizes of exposure areas on the peripheral retina during the color light adaptation. Experiment 2 was similar to the first, except for the inclusion of participants of varying ages. This was undertaken to address the issue that the loci of the achromatic points of older adults do not change despite their reduced neural sensitivity (Werner & Schefrin, 1993).

## Experiment 1

The aftereffect in the fovea was measured during color adaptation by exposing the peripheral retina to adaptive light to confirm whether chromatic adaptation, which occurs at post-retinal levels, displays rapid buildup and recovery. The aftereffect was measured as the location of the neutral point in color space measured in the fovea. Using this method, the effect of chromatic adaptation could be measured under conditions with little retinal photoreceptor adaptation.

### Purpose

The goal of this experiment was to understand the temporal characteristics of the part of chromatic adaptation that does not occur due to retinal photoreceptor adaptation. We measured the effect of the size of the retinal periphery involved in chromatic adaptation to determine the effect of the intensity of photoreceptor adaptation on its temporal characteristics. This enabled us to determine the extent to which the measured aftereffect of chromatic adaptation involves the adaptation of retinal photoreceptors.

### Methods

*Participants.* We recruited six female participants, ranging in age from 20 to 23 years, who had not previously participated in any psychophysical color experiments, and measured their neutral points before and after chromatic adaptation. Using Ishihara pseudo-isochromatic plates, all participants were confirmed to have normal color vision. Their visual acuity, including corrected visual acuity, was confirmed to be within the normal range.

*Experimental task.* The participants judged the chromaticity of a color patch presented on a monitor at every fixed time point through the integrating sphere filled with adaptation light.

**Figure 1.**
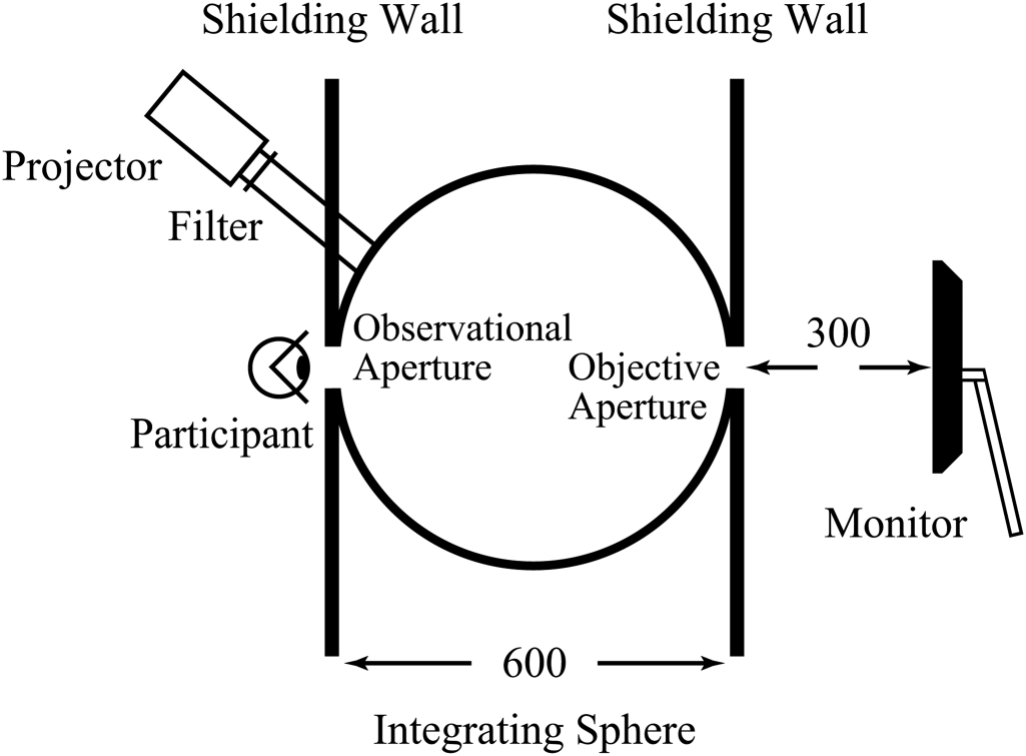
Apparatus arrangement. The participant observes test stimuli displayed on a monitor through an integrating sphere with two apertures on opposite sides and filled with yellow light from a projector. Two shielding walls are used to prevent light leakage.

**Figure 2.**
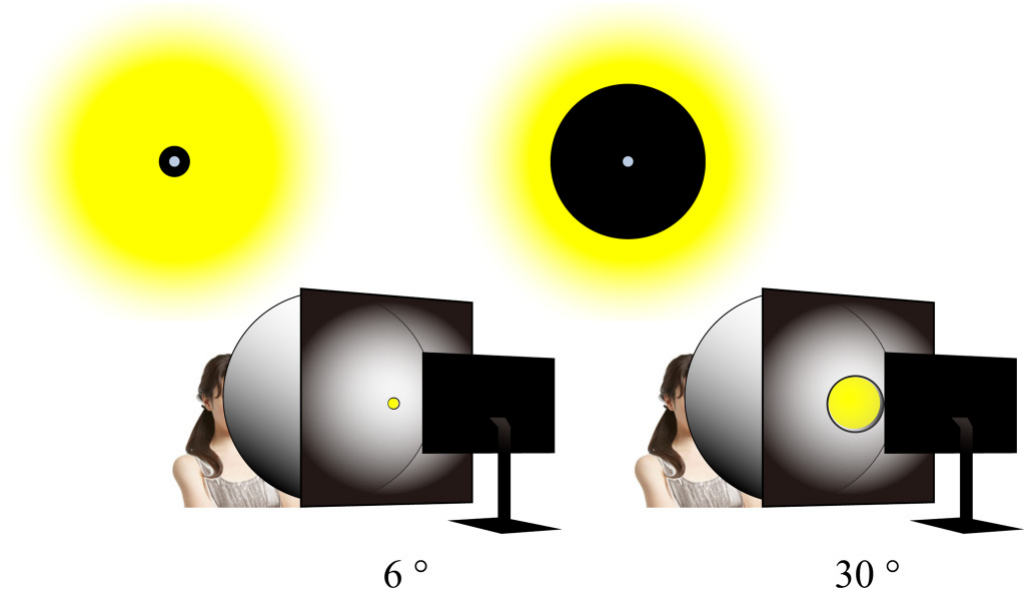
Two objective aperture sizes on the integrating sphere. Two sizes of objective apertures are used to study the effect of the retinal adaptation size and eccentricity of the adaptation area, thereby providing variety in the intensity of the adaptation in a controlled manner. The two concentric circles at the top of the figure indicate how the observer sees the stimulus in each condition.

*Apparatus and stimuli.* The adaptation stimulus was a yellow light projected from a halogen lamp through an optical sharp-cut filter (Fujifilm, Tokyo, Japan; SC˗52). As shown in Figure 1, the light was irradiated through the small holes of the entrance port of an integrating sphere that was internally coated with a white, diffusely reflective material and had a diameter of 600 mm. An excessively strong adaptation stimulus intensity could not be used because of issues concerning the ophthalmological effects on the experimental participants. We could not measure the effect of chromatic adaptation on the appearance of achromatic color in the fovea due to an overly weak adaptation light. Therefore, we changed the intensity of chromatic adaptation through the area size of the retina exposed using the adaptation light. We used two sizes of objective apertures (6° or 30° radius circular aperture, Figure 2) to confirm whether the size of the adaptation area on the retina affected the aftereffect of chromatic adaptation, as suggested by the magnitude of the neutral point shift, and to confirm whether the magnitude of chromatic adaptation depended on the distance between the adaptation area and the measurement area on the retina.

The observer’s head was positioned on a chin rest where they could observe the test stimuli presented on an organic electroluminescence display monitor, which was located on the opposite side of the integrating sphere and could be observed through two apertures, namely, an observation aperture and an objective aperture. The monitor (SONY, PVM-A250, Japan) was placed behind the objective aperture at a sufficient distance (300 mm) from the integrating sphere to prevent the monitor from being illuminated by light leaking from the integrating sphere with adaptive light. The monitor was controlled by PC (HP, Palo Alto, USA) and had a correlated color temperature of approximately 6,500 K, with a peak luminance of 88 cd/m^2^.

**Figure 3.**
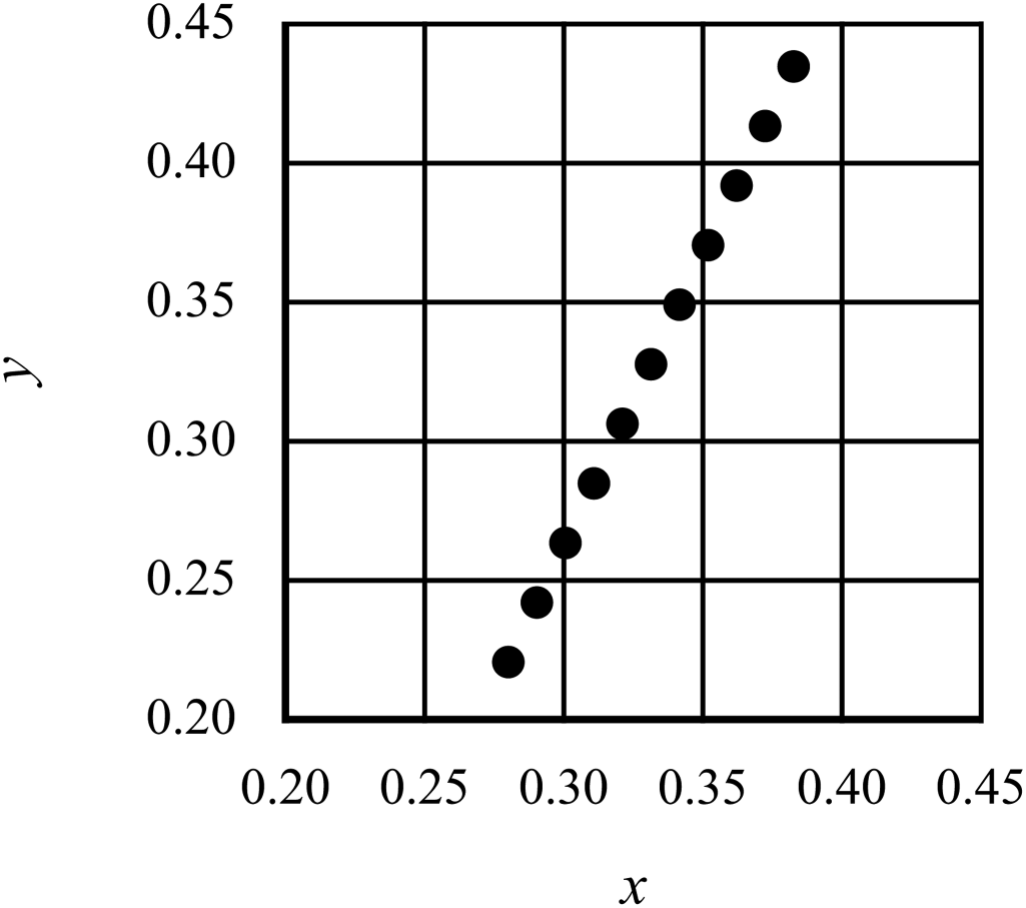
Chromaticity coordinates of the test stimuli of Experiment 1. The chromaticity of the test stimuli was changed on the tritan line close to the neutral point under daylight conditions to prevent any judgment caused by the brightness of the test stimulus, which is affected by the balance of the L and M cone output. The coordinates were those defined by the International Commission on Illumination (CIE) 1931 colorimetric system.

The test stimulus was a 2° diameter disk; its chromaticity was changed in 11 steps along the tritanopic confusion line (the tritan line; Figure 3) to avoid the influence of the direction of modulation and interactions between photoreceptors. These 11 chromaticity steps were isoluminated by flicker photometry adjustment for each observer. Participants observed the test stimulus through the apertures of the integrating sphere; their foveae were not exposed to, and therefore did not adapt to, the adaptation light. The color appearance for each observer can only be affected by chromatic adaptation caused by the exposure of the retina outside the fovea. All apparatuses were used in a darkened room.

*Procedure.* After 15 min of dark adaptation and subsequent critical flicker photometry to determine the series of the isoluminant stimuli set generated by flicker photometry for each participant, participants were exposed to white daylight (*x* = 0.322, *y* = 0.335) adjusted to the same luminance as that of the chromatic adaptation stimulus of the integrating sphere for 5 min. In the adaptation task, the participants observed a Japanese character changing randomly every second over a completely black background in the center of the organic light-emitting diode display monitor through the apertures of the integrating sphere filled with adaptation light and reported the appearance of a certain character on the screen. In this arrangement, the fovea may be slightly affected by adaptive light, but the effect of this adaptive light is considered negligible. Hence, the peripheral retinal influence can be ignored in the presentation of stimuli to the fovea. After daylight adaptation, the neutral point was measured four times using a one-up/one-down staircase procedure (the two-alternative forced-choice [2AFC]), in which the participant judged the color of the test stimulus on the same black background using two keys, depending on whether the test stimulus appeared bluish or yellowish. Since the stimulus sequence through the tritan line does not necessarily represent a completely achromatic color for the observer during chromatic adaptation, the experiment was conducted with a 2AFC method for only bluish or yellowish colors to measure the neutral point. After the neutral point was measured under daylight conditions, the participants were exposed to yellow light (*x* = 0.532, *y* = 0.458) with the integrating sphere following the same character detection task protocol, and the neutral point was measured four times every 5 min, for 20 min of continuous adaptation and a total of 16 trials. The observer continuously fixated on the changing character or the following test stimulus on a black background through the integrating sphere filled with yellow adaptation light during all the trials.

Before the experimentation, the participants received adequate training in reporting a color adjustment in trials similar to those used in the experiment. The chromaticity of adaptation light was measured and confirmed before and after the experiment. After the experiment, participants were asked about their observations or perceptions during the experiment.

The study protocol was approved by the ethics committees of the Shiseido Global Innovation Center and Joshibi University of Art and Design. Experiments adhered to the tenets of the Declaration of Helsinki and were performed after providing each participant with a thorough explanation of the study protocol, its underlying principles, and additional pertinent information regarding color vision testing. All participants provided written informed consent.

*Statistical analysis.* Data was analyzed using the Statistical Package for Social Sciences (IBM SPSS Statistics for Windows, Version 22, Armonk, NY: IBM Corp). The Kolmogorov–Smirnov test for normality and the equal variance test were applied to ensure that the data met the assumptions for the parametric tests. One- and two-way analyses of variance (ANOVA) were applied to determine significant differences in neutral points among aperture sizes or time points. Where significant differences were observed, a *t*-test was applied to determine where the differences lay. The resulting *p* values were Bonferroni corrected for multiple comparisons.

### Results

**Figure 4.**
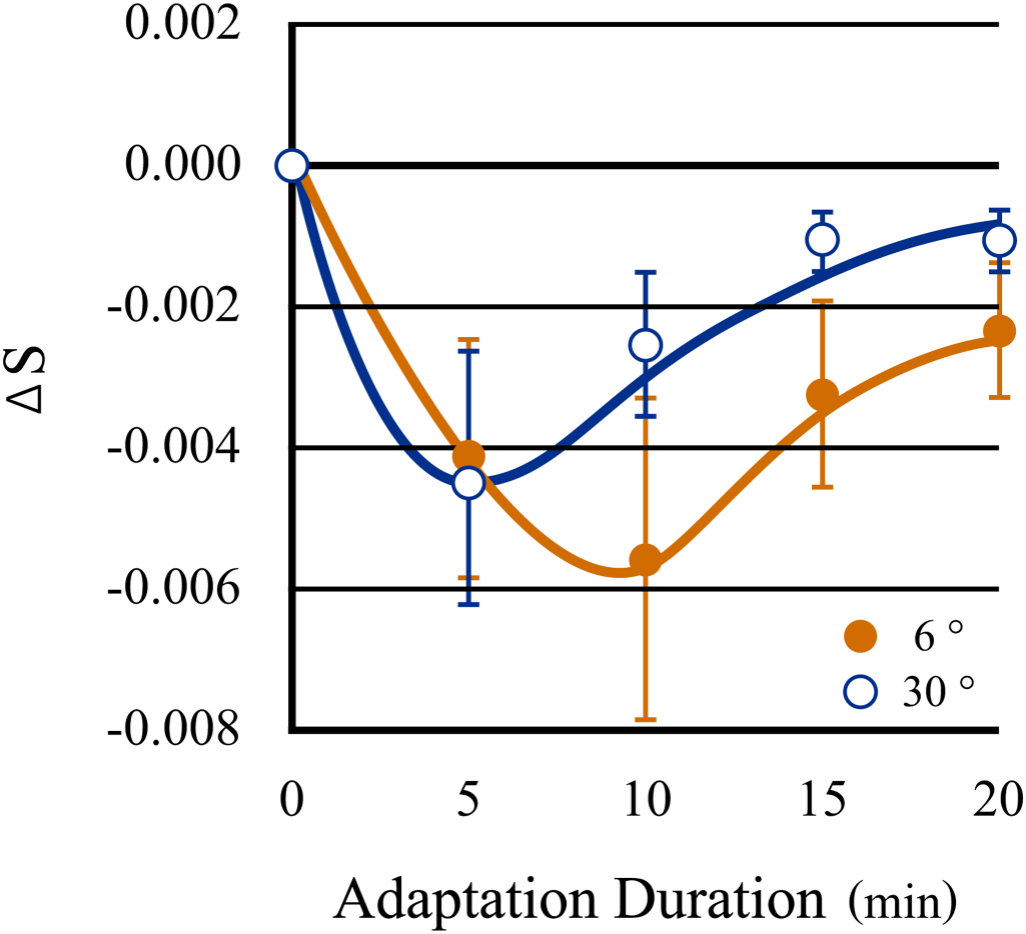
Transition distance of the neutral points along the S-axis (blue–yellow axis) as a function of the duration of chromatic adaptation (daylight = 0 min) recorded every 5 min. The neutral point shifted in the yellow direction initially because the sensitivity to yellow decreased immediately after the start of adaptation; subsequently, the neutral point gradually returned to the locus typical of daylight, regardless of the ongoing chromatic adaptation. The results obtained with the two aperture sizes are depicted by similar curves. Error bars indicate standard errors.

Each measurement of the neutral point measured as aftereffect was completed within 1 min (range: 20–60 s). However, the term “aftereffect” may not be completely accurate because the effects of color adaptation were measured during this experiment. The effect of chromatic adaptation on the appearance of color was termed the “aftereffect” in a previous study (Shimakura & Sakata, 2019). Therefore, this term was used for the cumulative effect of color adaptation in our study. The aftereffect of the chromatic adaptation to yellow light was analyzed as a transition of the neutral point on the MacLeod and Boynton chromaticity coordinates (MacLeod & Boynton, 1979). Data with very long reaction times and extremely large standard deviations (> 2σ) were excluded from the analysis for reasons of reliability. Figure 4 shows the transition of the neutral point recorded every 5 min during chromatic adaptation as a distance along the blue–yellow axis from the coordinate representing daylight adaptation (0 min). The abscissa in the figure shows the duration of the chromatic adaptation in minutes, and the ordinate shows the magnitude of the aftereffect as the distance of transition of the neutral point in the MacLeod and Boynton chromaticity coordinates. The time series transitions showed a quick increment and a slow decrement in the adaptation aftereffect; that is, the neutral point largely transitioned toward yellow in the very early stages of chromatic adaptation and then approached the neutral point of daylight adaptation regardless of the aperture size. As shown by the fitted curves, the data obtained from both objective apertures were consistent with a gamma distribution, typical for perceptual phenomena (Kolmogorov–Smirnov *p* > .05 for both aperture data; the best-fit parameters for the gamma distribution were: shape parameter [*k*] = 2.7; scale parameter [*θ*] = 4.9 for 6° *D* = 0.016, and *k* = 1.1, and *θ* = 8.6 for 30° *D* = 0.024).

Two-way ANOVA was used to examine the influences of aperture size and time course. The test confirmed that the shift in the neutral point by chromatic adaptation through the smaller 6° aperture was stronger than that through the larger 30° aperture (*F*[1, 173] =24.83, *p* < .001) after confirming the equality of variances by Levene’s test. Furthermore, it was confirmed that the time course had an effect (*F*[4, 173] = 13.38, *p* < .001); however, no interaction was significant (*F*[4, 173] = 1.80, *p* = .132). One-way ANOVA also revealed that the effect of time course in each aperture was significant (*F*[4, 90] = 8.54, *p* = .0001 at 6° aperture; *F*[4, 83] = 6.978, *p* = .0001 at 30° aperture). A *t*-test for multiple comparisons with Bonferroni correction confirmed that both results indicated similar behavior, namely, an initial increase was observed in the aftereffect 5 or 10 min after the start of chromatic adaptation, between min 0 and 5 (*p* < .01), between min 0 and 10 (*p* < .001), between min 0 and 15 (*p* < .02) at 6°, between min 0 and 5 (*p* < .001), and between min 0 and 10 (*p* < .02) at 30°; thereafter, the gradual decrement in the aftereffect was confirmed by one-way ANOVA (*F*[3, 72] = 5.27, *p* = .002 at 6°; *F*[3, 68] = 4.91, *p* = .004 at 30°) between min 5 and 20, and *t*-test confirmed the decrement between min 10 and 20 (*p* < .01) at 6°, between min 5 and 15 (*p* < .01), and between min 5 and 20 (*p* < .02) at 30°, while chromatic adaptation was ongoing.

### Discussion

As chromatic adaptation in the peripheral retina continued during the experiment in which the aftereffect was measured in the fovea, the aftereffects would have continued to increase if the aftereffect measured in the fovea accumulated the aftereffect of the peripheral area. However, the aftereffect began decreasing 5 to 10 min after the start of chromatic adaptation. Regardless of the aperture size, the aftereffects increased in the initial phase and decreased in the later phase, despite the ongoing chromatic adaptation in the peripheral retina. The fact that the aftereffect decreased despite the ongoing adaptation may indicate that the measured aftereffect was not provided solely by the accumulation of the adaptation of the peripheral retina until the end of the trials.

At 20 min after the start of adaptation, the neutral points had not returned to the initial daylight loci in either of the aperture size conditions. Therefore, the residual aftereffect may include an aftereffect due to continued chromatic adaptation in the peripheral retina. However, the aftereffect decreased gradually over time in both the aperture size conditions, indicating that the size of color adaptation of the periphery of the retina had little effect on the results. The transition of the neutral point, which represents the intensity of adaptation, showed the same shape but a different magnitude because of the size of the aperture on the integrating sphere, which defined the adaptation area size and the eccentricity on the retina. This finding points to the low possibility of the existence of a complex mechanism wherein both the retinal interaction and the post-retinal interaction affect the amount of adaptation.

The fact that the neutral point initially moved toward yellow and then returned to the approximate daylight point suggested a lag in the time course between the aftereffect and recalibration of chromatic adaptation, and it was not possible to compensate completely for color appearance under daylight within 20 min. In addition, the observers’ neutral points closely approximated those in daylight adaptation in this experiment, which might suggest cortical processes, rather than photoreceptors, in the human color compensatory mechanisms associated with long-duration adaptation.

Differences in aperture size changed the intensity of chromatic adaptation, which may have occurred because the retinal adaptation size was larger with the small aperture. However, the temporal progression of compensation was similar under both conditions. When the aperture size was large, that is, when the adaptation size on the retina was small, the degree of adaptation was less pronounced; however, the adaptation reached its peak early. Conversely, when the adaptation size on the retina was large and the adaptation was strong, the aftereffect was large; however, the time to reach the peak was longer. Thus, the time course and magnitude of chromatic adaptation showed a certain tradeoff, suggesting that recovery from stronger adaptation takes longer. The results under both aperture size conditions had similar temporal course transactions, which was an initial increase and then a decrease to approximately daylight levels. Therefore, we considered that the neural direct interactions on the retina in the 6° condition, if present, were negligibly small, as in the 30° condition.

## Experiment 2

Color light adaptation caused by sensitivity changes in the retinal cones develops and recovers in a very short time; in contrast, the age-related yellowing of the optics exposes our retinas to long-term yellow light, and long-term color adaptation may occur with aging. Therefore, the same protocol as that of Experiment 1 was used with participants of a relatively wide age range to determine whether the recalibrations observed in Experiment 1 could also be observed in relatively older adult participants.

### Purpose

This experiment investigated the effect of age on the compensation of color appearance in chromatic adaptation by measuring the transition of the neutral point under yellow light adaptation.

### Methods

**Figure 5.**
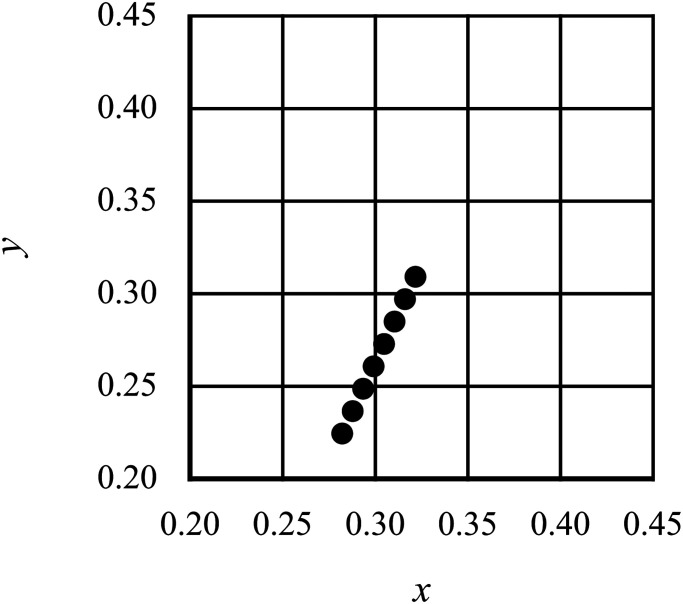
The International Commission on Illumination (CIE) chromaticity coordinates of the test stimuli in Experiment 2. The color of the stimulus was changed in eight steps on the tritan line.

The task, apparatus, and stimuli were the same as in Experiment 1, with the exception that the size of the aperture of the integrating sphere was only 6°, the test stimulus had five levels of luminance (66, 72, 80, 85, and 90 cd/m^2^), and the stimulus chromaticity was changed in eight steps along the tritan line (Figure 5). The experiment included 20 female participants in two groups, aged 55–75 years (*n* = 10) and 20–22 years (*n* = 10), with no history of ophthalmological surgery. All participants had a normal color vision and visual acuity confirmed with the same procedure as in Experiment 1. Written informed consent was provided by all the participants and the experiment adhered to the tenets of the Declaration of Helsinki. The study protocol of Experiment 2 was also approved by the ethics committees of the Shiseido Global Innovation Center and Joshibi University of Art and Design. The procedure was the same as in Experiment 1 with the exception that after five trials under daylight conditions, the participants were exposed to yellow light and their neutral points were measured five times every 10 min, for 70 min of continuous adaptation and a total of 35 trials.

### Results

**Figure 6.**
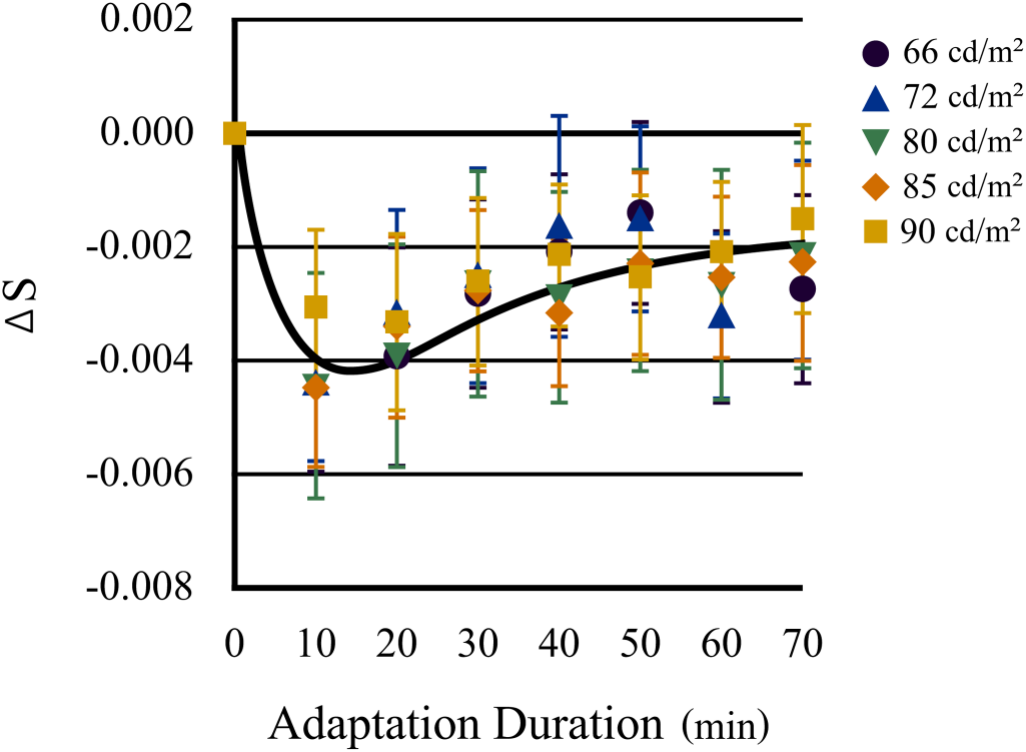
Time course of the transition of the neutral point along the blue–yellow axis after daylight adaptation in older eyes. The results for older eyes are broadly affected by the luminance levels of the test stimuli and display weak adaptation early during chromatic adaptation, followed by a slight shift toward the daylight neutral point. Error bars indicate standard errors.

The average transition of the neutral points along the tritan line before and after chromatic adaptation in older eyes is summarized in Figure 6. The duration of the chromatic adaptation is indicated by the abscissa, and the transition distance of the neutral points along the S-axis before and after adaptation is indicated by the ordinate. The color of each symbol indicates the luminance level of the test stimuli.

A one-way ANOVA confirmed the statistical significance of the main effect of luminance level for older eyes (*F*[4, 316] = 4.08, *p* = .003); however, the *t*-test did not reveal any significant difference between the duration of chromatic adaptation at each luminance level.

In older eyes, neutral points shifted from those under daylight (*F*[7, 343] = 6.53, *p* < .001) and a significant difference between daylight adaptation and each subsequent adaptation time point was confirmed by *t*-test (*p* < .001 for min 0–10; *p* < .003 for min 0–20; *p* < .02 for min 0–30; *p* < .03 for min 0–40; *p* < 0.01 for min 0–50; *p* < 0.05 for min 0–70); however, the significant differences between the S values of the neutral points at the peak of the aftereffect and at each subsequent adaptation time point could not be confirmed. The recovery of the neutral point observed at Experiment 1 was hardly observed at each timepoint of adaptation in the time span of this study. These results suggest that in older eyes, the aftereffects of chromatic adaptation, and thus chromatic compensation, were weak, notwithstanding the presence of any long-term adaptation.

**Figure 7.**
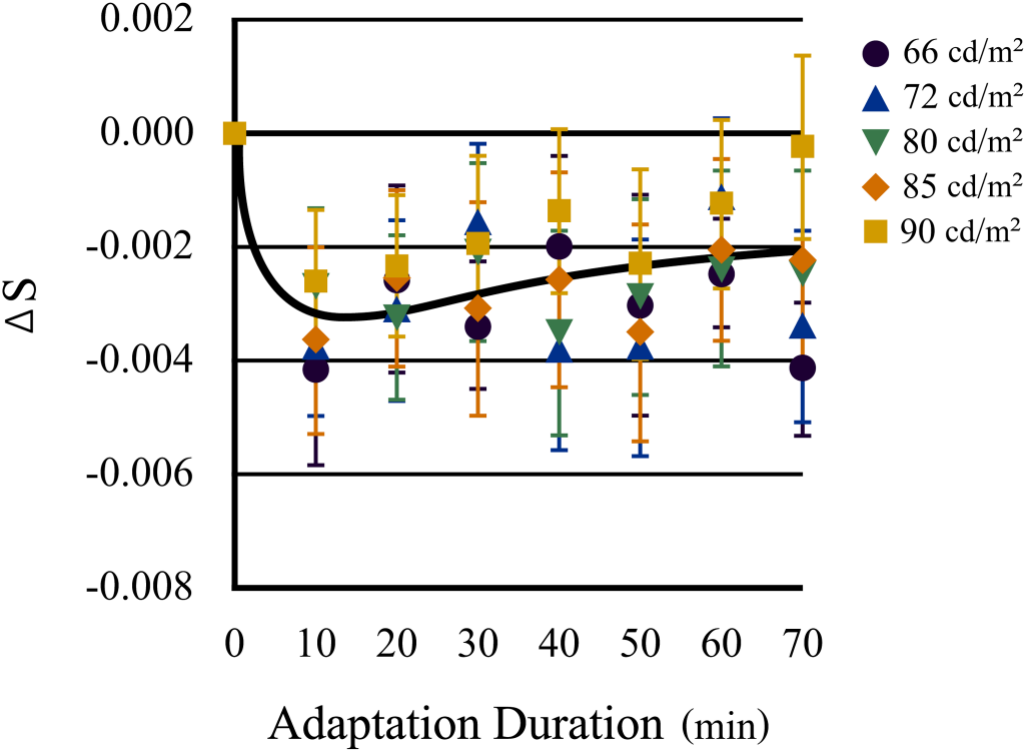
Transitions of the neutral points along the S-axis (blue–yellow axis) in younger eyes as a function of time of chromatic adaptation to yellow light (daylight = min 0). The color of each symbol shows the luminance level of the test stimuli. Neutral points in younger eyes show significant transition to yellow during the initial 10 min of chromatic adaptation and then gradually but significantly return to the same point as under daylight adaptation, despite the retina (except the fovea) being continuously exposed to the adaptation light. Error bars indicate standard errors.

Conversely, the younger eyes demonstrated no significant differences between luminance levels (*F*[4, 316] = 1.49, *p* = .205), whereas the neutral points in the first 10 min of chromatic adaptation differed significantly from those observed under daylight conditions (*p* < .001), as shown in Figure 7. In younger eyes, the magnitude of the shifts in color appearance due to chromatic adaptation shifted toward yellow (*F*[7, 343] = 11.03, *p* < .001) and reached a maximum after 10 min of adaptation. Thereafter, the loci of the neutral points approached those of daylight adaptation (*p* < .02 for min 10–40; *p* < .05 for min 10–50; *p* < .03 for min 10–60; *p* < .005 for min 10–70; *p* < .05 for min 20–50), but it did not return completely (*p* < .001 for min 0–20; *p* < .02 for min 0–30; *p* < .03 for min 0–40; *p* < .005 for min 0–60). Therefore, the data from the younger eyes typically exhibited distinct shifts in color appearance owing to chromatic adaptation, indicating more aftereffect and compensation.

A two-way ANOVA confirmed the statistical significance of the main effect of time course (*F*[7, 56] = 36.17, *p* < .0001); however, the difference between younger and older eyes was not significant (*F*[1, 8] = 0.969, *p* = .354). The post hoc paired *t*-tests (Bonferroni corrected) confirmed a significant difference in neutral point loci between older and younger eyes at 10 min after adaptation (*p* < .02) after confirming the equality of variances by Levene’s test. On the contrary, there was no significant difference in the neutral point between older and younger eyes in the daylight adaptation condition.

### Discussion

Since the peripheral part of the lens of the optical system of the eye is thin, the degree of color adaptation in this experiment was relatively similar between older and younger eyes. In the vicinity of the fovea used for the test trial, the yellowing concentration of the eye optical system in older adult eyes appeared to be more progressive than younger eyes. However, if the color compensatory mechanism of older adults is the result of chromatic adaptation owing to the progressive yellowing of the optical system over a long period, color compensation may perform more effectively as the yellowing progresses. Indeed, no difference was observed between the neutral points of younger and older eyes under daylight conditions. This fact suggests that white is perceived the same, regardless of the aging of the visual optics, revealing that a compensatory mechanism is in effect.

The neutral points of both older and younger eyes continued to shift during the experiment. Specifically, the shift in color appearance peaked at 10 min, after which it gradually decreased, approaching the neutral point observed under daylight at approximately 60 min. There may be several reasons why the transition of neutral points under daylight adaptation conditions was less pronounced in older than younger eyes, despite the overall similarity. The adaptation stimulus light reaching the retinal photoreceptors in older eyes could be less intense than that in younger eyes, not only because of the yellowing of the optical system but also because of the decrease in pupil diameter that occurs with aging. Thus, the magnitude of chromatic adaptation may be smaller in older than younger eyes. The photoreceptors of older eyes may also be less sensitive than those of younger eyes. Moreover, the participants in the older group may have already adapted to yellow light due to the yellowing of their optics over time.

These factors could explain why significant differences were observed between luminance levels in older eyes. While the aftereffect of chromatic adaptation through high pervious pale optics of younger eyes saturated, participants with older eyes could be more sensitive to the luminance of adaptation light, as if they were wearing sunglasses. The speculation that variation in the ocular optics density with age clearly reveals that the difference in luminance levels in the achromatic appearance is congruent with the experimental result, showing differences between luminance conditions observed in participants with older (55–75 years) eyes with a greater variance compared with those with younger (20–22 years) eyes with a smaller variance.

Conversely, the results revealed that the aftereffects of yellow adaptation were more robust in younger eyes than in older eyes. Specifically, as the level of yellowing was lower in the younger eyes, the magnitude of chromatic adaptation was larger and more obvious in younger eyes than older eyes, and the results may indicate a significant compensation of returning the neutral point to daylight. As the neutral point had returned to white under daylight not only among older eyes but also among younger eyes, younger eyes must also have a compensatory mechanism, despite their low or nonexistent levels of yellowing. If this compensatory mechanism was formed by long-term yellow adaptation caused by optical yellowing over the human lifespan, we might not have observed the compensation in younger eyes in such a short length of adaptation time, as shown in this experiment. We simulated the same compensation in participants without optical yellowing by a 1-h exposure to yellow light adaptation, and the result suggests that this compensation of chromatic adaptation may be the function of the cortical area.

## General Discussion

The buildup and recovery of adaptation, as suggested in a previous study (Tregillus & Webster, 2014), were confirmed in Experiment 1 by measuring the shift in the neutral point due to color adaptation in the peripheral retina. The experimental results showed that the chromatic adaptation mechanism, which may exist at post-retinal levels, recovered in 30 min to 1 h, despite the ongoing color adaptation. This result indicates that the relatively long-term chromatic adaptation, which may exist at the cortical level, does not build up and even if it does, rapidly recovers. This recovery of chromatic adaptation occurred not only in comparatively older adult eyes, whose retinas are thought to be constantly exposed to yellow light due to the high density of eye optics, but also in younger eyes, which are more transparent and have less color adaptation in daily life.

The method of chromatic adaptation examined in our experiments was peripheral adaptation; briefly, adaptation light was projected only on the peripheral area of the retina, and the fovea received negligible exposure. Therefore, the photoreceptors used to adjust the test stimuli to measure the shift in neutral point due to chromatic adaptation were hardly affected by the adaptation light. Subsequently, the shift in color appearance by chromatic adaptation in these experiments must have been caused not by the loss of sensitivity in the retinal photoreceptors, but by adaptation in higher neural mechanisms, which may reside in the visual cortex.

Naturally, the involvement of sensitivity loss in the photoreceptors of the fovea should not be neglected, considering that the eyeball has a structure similar to that of the integrating sphere. Considering the structure of the eyeball, adaptive light applied only to the periphery of the retina may have affected the photoreceptor cells in the fovea, even if only slightly. However, the aftereffects of photoreceptor adaptation are known to be highly ephemeral, typically lasting only a few seconds (Rinner & Gegenfurtner, 2000), and the duration of these experiments could be too long for photoreceptor adaptation, which is known to exhibit temporal characteristics different from those of the central components of adaptation to chromatic light (Shimakura & Sakata, 2019). This suggests that even if there was a slight effect of the adaptive light on the foveal photoreceptor cells, the results were due to the effects of a long-term adaptation mechanism, which is reported to have different temporal characteristics (Vincent et al., 2016; Belmore & Shevell, 2008, 2011). The duration of the chromatic compensation observed in our experiments could not be explained only by the photoreceptor contribution. In Experiment 2, even in older eyes, the duration of the peak and time course of the compensatory mechanism was the same as those in Experiment 1, not only confirming the reliability of the data but also suggesting central cortical processes, rather than the involvement of the retina (irrespective of retinotopy), in the compensatory mechanism of peripheral adaptation. The aftereffects and compensation of chromatic adaptation demonstrated in these experiments must be attributable to the cortical neural mechanism of color vision, and our results suggest that the compensatory mechanism of chromatic adaptation is one of the functions of long-term chromatic adaptation, which have been reported as the result of colored filter adaptation (Neitz et al., 2002).

The results obtained from these experiments showed compensation, that is, the recovery of chromatic adaptation during the continuous application of the adaptation light. As no daylight cue was available in the test stimuli, participants could not reference daylight white when perceiving the test stimuli in chromatic adaptation. The findings that the correct direction and magnitude of compensation in all participants suggest that daylight information is stored somewhere in our color vision mechanism. Our cortical mechanism of color vision may help to determine an achromatic point under daylight conditions and to refer to it throughout our lifespan because chromatic discrimination abilities depend on daylight (Mollon, 2006; Parry et al., 2008; Shimakura & Sakata, 2009; Danilova & Mollon, 2014).

Finally, we emphasize the importance of the recovery function during color adaptation observed in this study, which may have important implications in natural viewing conditions. For example, even in situations in which the surrounding environment is only a specific color light, such as sunset or underwater, we may be able to estimate the color appearance under daylight after a while. The interactions of color information processing between the peripheral visual field and the fovea demonstrated in this study are suggested because the central color of the visual field and the peripheral color have different effects on visual search performance (Nuthmann & Malcolm, 2016).

### Conclusion

Our color vision mechanism is tuned to see color under daylight, and this ability serves as a compensatory function not only after but also during color adaptation in response to long-term exposure to colored light. The information on the appearance of colors under daylight is stored in our color vision mechanism, and it appears to continue to readjust the appearance of colors even if the light reaching the retina becomes yellowish in old age.

### Limitations

One limitation of this study was that we were unable to completely reproduce the compensation of chromatic adaptation under daylight. Longer and more intense chromatic adaptation may enable us to approximate complete adaptation, possibly throughout the human lifespan. This study revealed that the color vision mechanism continues to compensate for various visual inputs throughout our lifespan to yield the perception of apparent color under daylight.

Another limitation was that all the participants in this study were females, and there are some reports on sex-related differences in human color vision (Murray, et al., 2012). Although this difference does not appear large, it is nonetheless important to confirm if similar experimental results are observed in male participants.
